# Explaining differences in self-focused and other-involved public health preventive behaviors between the US and China: the role of self- construal and health locus of control

**DOI:** 10.3389/fpubh.2024.1321506

**Published:** 2024-02-22

**Authors:** Wenjing Pan, Wang Liao, Bo Feng, Siyue Li

**Affiliations:** ^1^College of Journalism and Communication, Renmin University of China, Beijing, China; ^2^Department of Communication, University of Washington, Seattle, WA, United States; ^3^Department of Communication, University of California, Davis, Davis, CA, United States; ^4^College of Media and International Culture, Zhejiang University, Hangzhou, China

**Keywords:** health locus of control, health intervention, public health, self-construal, cultural differences

## Abstract

**Background:**

This study examined national similarities and differences in people's engagement in health preventive behaviors during a public health crisis, as well as investigated the underlying individual-level psychological mechanisms. A conceptual distinction was made between self-focused and other-involved preventive behaviors in response to public health crises.

**Method:**

Two cross-sectional surveys were conducted in the United States (*N* = 888) and China (*N* = 844) during the early stage of the COVID-19 pandemic. Hayes' PROCESS was utilized to assess national differences in seven preventive behaviors, along with the mediating effects of self-construal and health locus of control.

**Results:**

The results showed that American participants reported greater engagement in self-focused preventive behaviors than Chinese, whereas Chinese participants reported greater engagement in other-involved preventive behaviors than Americans. Chinese participants also engaged more in other-involved than self-focused preventive behaviors. Self-construal and health locus of control partially explained the observed differences in engagement in preventive behaviors.

**Discussion:**

This study introduces a culture-sensitive approach to provide insights for crafting communication interventions that can enhance the effectiveness of health campaigns in the context of a public health crisis.

## 1 Introduction

Over the past century, the world has been witnessing several waves of public health crises, with the recent coronavirus disease (COVID-19) being one of the most prominent one in human history (1). The battle against this public health crisis has revealed national differences in both the impact of the virus and approaches to dealing with it. For example, countries that are conventionally considered individualist societies tend to have more COVID-19 cases than collectivist societies (2). People from countries with a culture less tolerant of uncertainty and ambiguity also tend to have fewer social gatherings, which might have helped control a public health crisis in those countries (3).

Although the abovementioned studies explain the national differences through national cultures, it is crucial to investigate individual-level psychological mechanisms to guide behavioral interventions and communication campaigns in tackling a public health crisis. This study addresses such mechanisms by surveying people who lived in the United States and China during the pandemic regarding their engagement in preventive behaviors against the COVID-19 pandemic. Public crises, such as the pandemic, often present both independent, individual risks and interdependent, communal risks, thus requiring not only self-protection but also collective actions of a larger community (4). In response to both individual and communal risks, we first distinguish between two types of preventive behaviors: *self-focused* and *other-involved* preventive behaviors. The former refers to preventive behaviors performed in private settings, while the latter highlights the interdependence among individuals as such preventive behaviors require social monitoring or cooperation from others. Examining these two types of preventive behaviors helps us understand individual actions in relation to others and better tap into the communal nature of most public health crises.

To illuminate the psychological mechanisms underlying self-focused and other-involved preventive behaviors, we further investigate two culturally linked psychological traits: self-construal and health locus of control. Self-construal refers to the extent to which individuals see themselves as independent or interdependent in relation to others and is a relatively stable aspect of an individual's identity (5). Health locus of control refers to how people attribute responsibilities for their health to internal factors, powerful others, and chance (6). In the context of the current study, we specifically explore whether people believe that their health during the pandemic is controlled by their own actions, such as wearing masks or social distancing (i.e., internal factors), by health authorities or government mandates (i.e., powerful others), or by chance. As will be discussed later, both self-construal and health locus of control are highly influenced by culture. They not only help explain the national differences in self-focused and other-involved preventive behaviors but also provide guidance for designing personalized health interventions.

In brief, the objective of this study is to examine similarities and differences between American and Chinese people's engagement in self-focused and other-involved public health preventive behaviors. To achieve this goal, we first explicate the two types of preventive behaviors and discuss how engagement in these behaviors might differ between Americans and Chinese. We then explore how self-construal and health locus of control account for the national differences in engagement in the two types of preventive behaviors. Next, we report a survey study designed to investigate our hypotheses. Implications of the findings are discussed in relation to global recovery and health interventions aimed at combating a public health crisis.

## 2 Literature review

### 2.1 A categorization of preventive behaviors during a public health crisis

Previous research highlights how specific preventive behaviors during a public health crisis differ across nations (e.g., wearing masks; (7), yet it overlooks the similarities and differences across various health preventive behaviors. We argue that preventive behaviors can be theoretically categorized as either *self-focused* or *other-involved*. Self-focused preventive behaviors are those that are often performed in relatively private settings that involve minimal monitoring, social interaction, or cooperation from others. Washing hands and disinfecting surfaces, as recommended by the Centers for Disease Control and Prevention (CDC) in both China and the United States, are examples of self-focused preventive behaviors. In contrast, other-involved preventive behaviors are typically performed in public settings and can be easily observed and monitored by the public. The effectiveness of other-involved preventive behaviors also depends on others' cooperation and implies a need to understand a public health crisis as an interdependent, communal risk. Seen in this light, wearing a mask (typically performed in a social setting), covering nose and mouth when sneezing and coughing, keeping social distance, staying home (the opposite of going outside), and avoiding public transportation (the opposite of taking public transportation) can all be considered other-involved preventive behaviors.

While the abovementioned preventive behaviors all serve the purpose of preventing the transmission of the disease, societies with different cultural backgrounds may exhibit different acceptance of self-focused or other-involved preventive behaviors. In particularly, individualistic societies may favor self-focused preventive behaviors over other-involved behaviors because individualistic cultures typically endorse personal choice and individual interest (8). Self-focused behaviors, in essence, protect one's own vital interests, whereas the other-involved behaviors likely impose constraints on one's autonomy. Similarly, collectivist societies value collective interests and social norms (8) and thus are likely to encourage other-involved preventive behaviors more than self-focused preventive behaviors. Given that the United States and China are conventionally considered individualist and collectivist societies, respectively (9), we postulate that people from these two societies would engage differently in the two types of preventive behaviors. These differences should be manifested both between the two nations and within each of them, as postulated below.

H1a: Compared to the Chinese, Americans will be more frequently engaged in self-focused preventive behaviors (e.g., washing hands and disinfecting surfaces).
H1b: Compared to Americans, Chinese will be more frequently engaged in other-involved preventive behaviors (e.g., wearing a mask, covering nose and mouth when sneezing and coughing, keeping social distance, staying home, and avoiding public transportation).
H2a: Americans will be more frequently engaged in self-focused preventive behaviors than other-involved preventive behaviors on average.
H2b: Chinese will be more frequently engaged in other-involved preventive behaviors than self-focused preventive behaviors on average.

### 2.2 Psychological traits and preventive behaviors

While differences in self-focused and other-involved preventive behaviors can exist at the national level, both between and within the United States and China, we need to delve into the underlying individual-level psychological mechanisms to inform potential behavioral interventions and communication campaigns. As discussed in the following paragraph, two psychological traits—self-construal and health locus of control—are especially relevant.

#### 2.2.1 Self-construal and preventive behaviors

Self-construal is defined as “a constellation of thoughts, feelings, and actions concerning one's relationship to others, and the self as distinct from others” [(10), p. 581]. Prior research has documented two major forms of self-construal: independent and interdependent self-construal (5). An independent self-construal views the self as an autonomous person (5). Individuals with an independent self-construal tend to consider their personal desires, preferences, attributes, and abilities central and important to their own identities. In contrast, an interdependent self-construal defines the self as a flexible and variable unit that emphasizes connections with others and social contexts (10). Individuals with an interdependent self-construal tend to act more based on the expectations of others and social norms than their personal desires (11).

In the context of a public health crisis, independent and interdependent self-construal likely play different roles in an individual's engagement in self-focused and other-involved preventive behaviors. Research shows that people from China typically score higher in measures of interdependent self-construal, whereas people from the United States tend to score higher in independent self-construal (12, 13). Being more relationship-concerning and norm-abiding, people with an interdependent self-construal could be more motivated to engage in other-involved preventive behaviors, because such behaviors are likely to be perceived as a part of the collective efforts to battle infectious diseases that spread across a community. However, interdependent self-construal does not necessarily preclude self-focused preventive behaviors, as such behaviors may be perceived as protecting both themselves and others due to the awareness of the communal risks in a public health crisis and the interdependence between self and others. Therefore, we expect interdependent self-construal to have a positive impact on engagement in both self-focused and other-involved preventive behaviors.

H3: Individuals' interdependent self-construal will be positively associated with their engagement in (a) self-focused preventive behaviors and (b) other-involved preventive behaviors.

Independent self-construal may influence engagement in preventive behaviors differently. Individuals with high independent self-construal focus on autonomy and prioritize personal goals over group goals (11). Thus, they may be more motivated toward self-focused preventive behaviors, which closely align with their own interests. In contrast, other-involved preventive behaviors entail concerns over others' interests and sometimes may work against one's self-interest. For instance, staying home could protect oneself and others from infections, but this action also restricts one's freedom of movement and can cause inconveniences. Supporting this idea, research has found that people with a high degree of independent self-construal were less interested in keeping social distance (14) and climate-friendly actions that involve personal sacrifice (15), which are both other-involved preventive behaviors. Therefore, we expect independent self-construal to impact self-focused and other-involved preventive behaviors in opposing ways, as postulated below.

H4: Individuals' independent self-construal will be (a) positively associated with their engagement in self-focused preventive behaviors but (b) negatively associated with their engagement in other-involved preventive behaviors.

#### 2.2.2 Health locus of control and preventive behaviors

People from different countries may also vary in their health locus of control and, consequently, their engagement in self-focused and other-involved preventive behaviors. The concept of health locus of control can be traced back to Rotter (16) social learning theory, which differentiates individuals' attribution of a certain behavior to internal factors (i.e., events that are consequences of individuals' own actions) and external factors (i.e., events that are unrelated to individuals' actions). With respect to health-related behaviors, Wallston et al. (17) argue that the twofold conceptualization can be elaborated as follows: in addition to control by internal factors (i.e., health is the result of one's own actions), control by external factors can be further divided into two subcategories: control by powerful others (e.g., doctors, physicians, and health institutions) and control by chance (i.e., health is due to fate or chance).

Different dimensions of national culture (9) can influence the three loci of control. Regarding control by internal factors, people from individualist societies are more likely to perceive themselves as having control over their health compared to those from collectivist societies (18, 19), which is partly because individualistic countries value self-reliance and react strongly to a loss of personal control (20). For control by powerful others, another cultural dimension—power distance—is highly relevant. Power distance is “the extent to which the less powerful members of organizations and institutions accept and expect that power is distributed unequally” [(21), p. 9]. Countries with higher levels of perceived power distance tend to view control by powerful others or authority figures as more acceptable or positive (20). In public health contexts, the most relevant powerful others are health authorities, and therefore, a culture with high power distance may enhance its people's sense of health locus of control by these authorities (22). Finally, with respect to control by chance, the cultural dimension of indulgence is relevant. Indulgence (vs. restraint) refers to the gratification vs. control of the basic human desires to enjoy life (21). Individuals from indulgence-oriented societies tend to place greater importance on leisure and live an unrestrained and pleasure-seeking lifestyle; in contrast, individuals from restraint-oriented societies tend to view leisure as less important and restrain from hedonic lifestyles (23). Accordingly, research has shown that people living in restraint-oriented societies tend to pay more attention to their health [e.g., choosing healthier meals; (20)], irrespective of societal economic development. In contrast, people from indulgence-oriented societies tend to engage more in smoking and are less likely to adopt a healthy diet (20), suggesting that they are more likely to leave their health to chance or fate.

The United States and China are usually positioned at the opposite ends of the spectrums for the three cultural dimensions mentioned above (21). The United States is generally characterized by high individualism, low power distance, and indulgent lifestyles, while China exhibits high collectivism, high power distance, and restrained lifestyles. Accordingly, we expect that Americans and Chinese would differ in the three types of health locus of control, as postulated below.

H5: Americans will perceive a higher level of health locus control by (a) internal factors and (b) chance but a lower level of control by (c) powerful others compared to Chinese.

At the individual level, the three types of health locus of control then differently influence the self-focused and other-involved preventive behaviors. In terms of control by powerful others, previous research has consistently found that this health locus of control promotes compliance with health authorities (24). For example, patients tend to engage in health monitoring behaviors due to the fear of violating their doctors' recommendations (6). College students also show more willingness to use health apps and online trackers (25) and to reduce drug use due to their compliance with directives from health authorities (26). Because both self-focused and other-involved preventive behaviors are typically recommended by public health authorities, for example, the CDC guidance for COVID-19 prevention in the United States and China, we expect people with high levels of control by powerful others to comply with both types of preventive behaviors, as postulated below.

H6: Individuals' perceived health locus of control by powerful others will be positively associated with their engagement in (a) self-focused and (b) other-involved preventive behaviors.

With respect to control by chance, this health locus of control generally reduces people's engagement in preventive behaviors (17). Such a sense implies that a person's health depends on fate or chance, thus reducing the motivation to exert control over one's own health. The sense of control by chance was even found to encourage health-threatening behaviors, such as smoking, alcohol consumption, drug use, and avoiding a healthy diet (20, 26). In a public health crisis, where both self-focused and other-involved preventive behaviors would concern one's own health and require control over one's own behaviors, we expect people with higher levels of control by chance to be less likely to engage in either type of preventive behaviors, as postulated below.

H7: Individuals' health locus of control by chance will be negatively associated with their engagement in (a) self-focused and (b) other-involved preventive behaviors.

Finally, regarding control by internal factors, we argue that it can promote self-focused preventive behaviors but discourage other-focused preventive behaviors, in contrast to the uniformly positive or negative effects of the other two types of health locus control. By definition, control by internal factors entails a belief in one's self-control over their health condition (6). Such self-control is required for self-focused preventive behaviors, as they are performed in private settings that involve minimal monitoring from others. Therefore, a greater sense of control by internal factors will better motivate self-focused preventive behaviors. At the same time, individuals with a greater sense of health locus of control by internal factors often pay closer attention to their own health condition (6) and thus are more likely to engage in self-focused preventive behaviors, thereby protecting themselves (e.g., HIV preventive behaviors, (27); health-promoting prenatal behaviors, (28); dental care, smoking cessation, and flu shot, (6).

However, the sense of control by internal factors may discourage other-involved preventive behaviors. The theory of planned behavior (29) suggests that the successful adoption of a healthy behavior requires individuals to have perceived control over the recommended behavior. When the efficiency of the recommended behaviors depends on the implementation by not only oneself but also others, as in the case of other-involved preventive behaviors, people will consider themselves to have less control (over others' behaviors) and thus be less motivated to perform these recommended behaviors. Moreover, as mentioned above, individuals with higher levels of health locus of control by internal factors typically focus on their own health conditions (6). Given a person's limited cognitive resources, these individuals are likely to be preoccupied with attention to their own health conditions and self-focused preventive behaviors, leaving other-involved preventive behaviors to be less attended to.

Taken together, we hypothesize that the health locus of control by internal factors has opposite effects on the two types of preventive behaviors, as follows.

H8: Individuals' health locus of control by internal factors will be (a) positively associated with their engagement in self-focused preventive behaviors but (b) negatively associated with their engagement in other-involved preventive behaviors.

## 3 Methods

### 3.1 Participants and procedure

This study surveyed people (above 18 years old) from both the United States (*N* = 888; 47.5% female participants; 18–74 years old, *M* = 37.00, *SD* = 11.94) and China (*N* = 844; 55.6% female participants;18–64 years old, *M* = 31.71, *SD* = 8.40) in April 2020. Participants in the United States were recruited from Amazon Mechanical Turk (https://mturk.com) and were paid USD$ 0.75 each, while Chinese participants were recruited from Sojump (wjx.cn) and were each paid RMB¥ 3.5 (Chinese yuan, roughly the same as the amount paid to American participants based on currency rate).

We constructed the questionnaire first in English and then translated it into Chinese. To ensure the accuracy and appropriateness of the translation, a research assistant who is proficient in both English and Chinese independently back-translated the Chinese questionnaire into English. The research assistant did not read the English version before conducting the translation. The back-translated version was then compared with the English version, and any deviation in meanings was addressed by revising and re-translating the Chinese questionnaire (see Supplementary Table S3 for the final questionnaire items in both languages).

### 3.2 Measures

#### 3.2.1 Engagement in preventive behaviors

We measured seven preventive behaviors recommended by the WHO and the CDC in both the United States and China, using a 5-point scale (*1* = *never, 2* = *sometimes, 3* = *about half the time, 4* = *most of the time, and 5* = *always*). For the American sample, the items were prefaced with the question “[o]ver the past month [i.e., March 2020], how often have you engaged in the following practices to minimize the risk of contracting the coronavirus (COVID-19)?” Since the outbreak was first identified in China and the infected cases peaked around February 2020, we prefaced the same items with “[d]uring February this year,” for the Chinese sample.

Two preventive behaviors are considered as *self-focused*, namely, “washing hands” (*M*_US_ = 4.34, *SD*_US_ = 0.90; *M*_China_ = 4.11, and *SD*_China_ = 0.84) and “cleaning and disinfecting frequently touched surfaces” (*M*_US_ = 4.01, *SD*_US_ = 1.06; *M*_China_ = 3.56, and *SD*_China_ = 1.15). The other five preventive behaviors are *other-involved*, namely, “wearing face masks” (*M*_US_ = 3.38, *SD*_US_ = 1.49; *M*_China_ = 4.88, *SD*_China_ = 0.41), “covering nose and mouth when sneezing and coughing” (*M*_US_ = 4.38, *SD*_US_ = 0.91; *M*_China_ = 4.37, *SD*_China_ = 0.81), “keeping social distance” (*M*_US_ = 4.33, *SD*_US_ = 0.87; *M*_China_ = 4.51, *SD*_China_ = 0.68), “staying home” (*M*_US_ = 4.18, *SD*_US_ = 0.85; *M*_China_ = 4.39, *SD*_China_ = 0.69), and “avoiding public transportation” (*M*_US_ = 4.51, *SD*_US_ = 0.85; *M*_China_ = 4.42, *SD*_China_ = 0.88).

#### 3.2.2 Independent and interdependent self-construal

Participants' independent and interdependent self-construal were measured using a simplified version of the self-construal scale (12) on a 7-point Likert scale (*1* = *strongly disagree; 7* = *strongly agree*). The subscale for independent self-construal includes five items, such as, “[m]y personal identity, independent from others, is important to me” and “I prefer to be self-reliant rather than dependent on others.” The average scale score was 5.44 (*SD* = 0.93 and Cronbach's α = 0.84) in the American sample and 5.28 (*SD* = 0.91 and Cronbach's α = 0.72) in the Chinese sample. The subscale for interdependent self-construal includes another five items, such as “[m]y relationships with my friends and family are more important than my personal accomplishments” and “I try to meet demands of my group, even if it means controlling my own desires.” The average scale score was 4.99 (*SD* = 1.00, Cronbach's α = 0.78) in the American sample and 5.40 (*SD* = 0.79, Cronbach's α = 0.68) in the Chinese sample. See Supplementary Table S3 for all scale items.

#### 3.2.3 Health locus of control

A modified scale based on Norman et al. (30) was used to measure the three dimensions of the health locus of control on a 7-point scale (*1* = *strongly disagree; 7* = *strongly agree*). The health locus of control by internal factors was assessed with three items (e.g., “[i]f I fall ill with the virus, I believe I can recover on my own without going to the hospital”). The average scale score was 5.22 (*SD* = 0.32 and Cronbach's α = 0.73) in the American sample and 3.73 (*SD* = 1.00 and Cronbach's α = 0.60) in the Chinese sample. The health locus of control by powerful others was measured with three items (e.g., “[i]f I fall ill with the virus, I believe the health care system will help me recover”). The average scale score was 4.92 (*SD* = 1.12 and Cronbach's α = 0.78) in the American sample and 5.73 (*SD* = 0.89 and Cronbach's α = 0.66) in the Chinese sample. The health locus of control by chance was measured by four items, for example, “[i]t seems as if my health mostly depends on sheer coincidence.” The average scale score was 3.89 (*SD* = 1.42 and Cronbach's α = 0.82) in the American sample and 2.39 (*SD* = 0.99 and Cronbach's α = 0.68) in the Chinese sample. See Supplementary Table S3 for all scale items.

### 3.3 Data analysis

**Figure 1:**
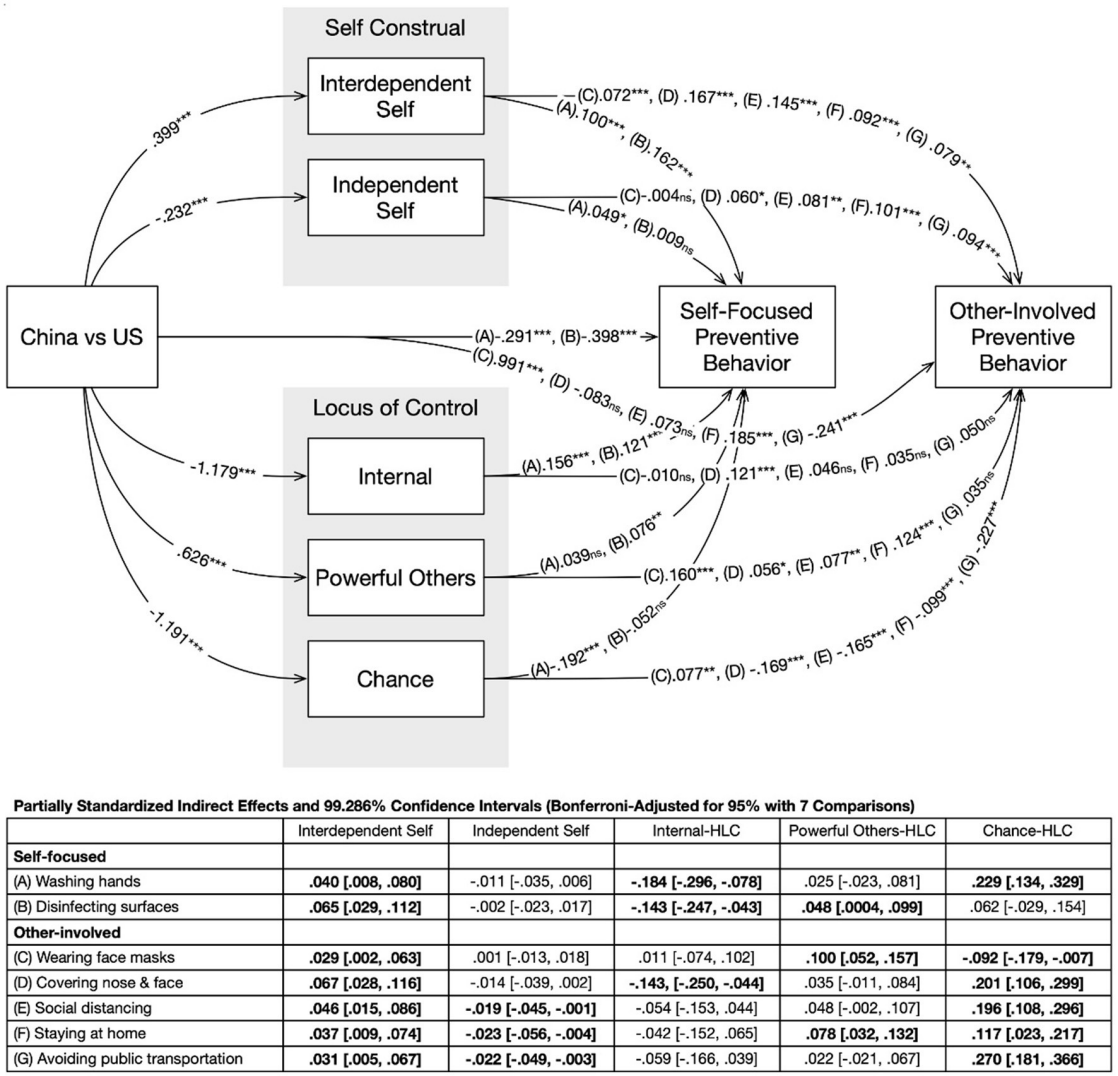
Path diagram and standardized coefficients. Coefficients for the seven preventive behaviors were listed on each path, following the order in the in-plot table. Significant indirect effects were bolded in the in-plot table. The following demographics are controlled: sex, age, education level, household income, marital status, and employment status. All significant tests were based on Bonferroni-adjusted *p*-values for seven comparisons. ^*^p < 0.05, ^**^p < 0.01, ^***^p < 0.001, and ns, non-significant.

We utilized Hayes (31) PROCESS to assess national differences in the seven preventive behaviors, along with the mediating effects of the two types of self-construal and three types of health locus of control, which are included as five parallel mediators. To examine these national differences in more detail, we tested all hypotheses except for H2(a-b) separately for each of the seven behaviors, with the *p*-values adjusted for seven comparisons using Bonferroni's method. Participants' sex, age, marital status, education level, income level, living condition, and employment status were included as covariates to control for potential confounding effects due to associations between demographics and preventive behaviors against COVID-19, which was found in previous research [e.g., (32)]. H2(a-b) involves within-subject comparisons and was tested using paired sample *t*-tests. Descriptive statistics of the key variables are included in Table 1 (see Supplementary Tables S1, S2 for summaries of the demographics of the two samples). The models and standardized estimates for all hypotheses, except for H2(a-b), are illustrated in Figure 1, along with bootstrapped 99.286% confidence intervals (CIs) (i.e., the Bonferroni-adjusted 95% CIs for seven comparisons, with 5,000 re-samples) for the indirect effects. The datasets, computer codes for the analyses, and all outputs can be found at https://osf.io/qae9k/?view_only=8cbbcb47c3a4441e8f366f1bad204ee3.

**Table 1:** Descriptive statistics of the key variables.

	**Americans (*****N*** = **888)**		**Chinese (*****N*** = **844)**	
	**Mean**	**SD**	**Mean**	**SD**
**Self-focused preventive behavior**				
Washing hands	4.34	0.90	4.11	0.84
Cleaning and disinfecting frequently touched surfaces	4.01	1.06	3.56	1.15
**Other-focused preventive behavior**				
Covering nose and mouth when sneezing and coughing	4.38	0.91	4.37	0.81
Keeping social distance	4.33	0.87	4.51	0.68
Staying at home	4.18	0.85	4.39	0.69
Avoiding public transportation	4.51	0.85	4.42	0.88
Wearing a face mask	3.38	1.49	4.88	0.41
**Self-construal**				
Interdependent	4.99	1.00	5.40	0.79
Independent	5.44	0.93	5.28	0.91
**Health locus of control**				
Internal	5.22	1.04	3.73	1.00
Powerful others	4.92	1.12	5.73	0.89
Chance	3.89	1.42	2.39	0.99

## 4 Results

H1(a) predicted that Chinese participants would engage less in self-focused preventive behaviors but (b) more in other-involved preventive behaviors than American participants. Supporting H1(a), regression analyses revealed that, compared to Americans, Chinese participants reported less engagement in the two self-focused behaviors, namely, washing hands with *b* = −0.17, *t*_(1723)_ = −2.81, and *p*_*adj*_ = 0.04 and cleaning and disinfecting frequently touched surfaces with *b* = −0.35, *t*_(1724)_ = −4.74, and *p*_*adj*_ < 0.001. Regarding H1(b), Chinese participants reported more engagement in three out of the five other-oriented behaviors, namely, wearing face masks with *b* = 1.36, *t*_(1724)_ = 18.53, and *p*_*adj*_ < 0.001; keeping social distance with *b* = 0.31, *t*_(1724)_ = 5.95, and *p*_*adj*_ < 0.001; and staying home with *b* = 0.29, *t*_(1724)_ = 5.55, and *p*_*adj*_ < 0.001, compared to Americans. The national differences in covering nose and mouth when sneezing and coughing (*p*_*adj*_ = 0.98) and avoiding public transportation (*p*_*adj*_ = 0.99) were not statistically significant. Therefore, H1(a) was supported while H1(b) was partially supported.

H2(a) predicted that American participants would engage more in self-focused rather than other-involved preventive behaviors, and H2(b) predicted that Chinese participants would engage more in other-involved than self-focused preventive behaviors. Paired-sample *t*-tests revealed that American participants did not differ in terms of their engagement in self-focused preventive behaviors (*M* = 4.18 and *SD* = 0.84) and other-involved preventive behaviors (*M* = 4.15 and *SD* = 0.66), *t*_(887)_ = 0.92 and *p* = 0.36. However, Chinese participants engaged more in other-involved preventive behaviors (*M* = 4.51 and *SD* = 0.39) than self-focused preventive behaviors (*M* = 3.84 and *SD* = 0.87), *t*_(843)_ = 24.61 and *p* < 0.001. Thus, H2(a) was not supported, but H2(b) was.

H3(a) and H3(b) postulated that interdependent self-construal should encourage both self-focused and other-involved preventive behaviors. Regression analyses showed that interdependent self-construal was positively associated with washing hands with *b* = 0.09, *t*_(1720)_ = 3.79, and *p*_*adj*_ = 0.01 and cleaning and disinfecting frequently touched surfaces with *b* = 0.18, *t*_(1720)_ = 5.86, and *p*_*adj*_ < 0.001, thereby supporting H3(a). Similarly, interdependent self-construal was positively associated with wearing face masks with *b* = 0.19, *t*_(1720)_ = 6.13, and *p*_*adj*_ < 0.001; covering nose and mouth when sneezing and coughing with *b* = 0.15, *t*_(1720)_ = 6.66, and *p*_*adj*_ < 0.001; keeping social distance with *b* = 0.10, *t*_(1720)_ = 4.78, and *p*_*adj*_ < 0.001; staying home with *b* = 0.08, *t*_(1720)_ = 3.94, and *p*_*adj*_ < 0.001; and avoiding public transportation with *b* = 0.06, *t*_(1720)_ = 2.68, and *p*_*adj*_ = 0.049, thereby supporting H3(b).

H4(a) and H4(b) postulated that independent self-construal will encourage self-focused preventive behaviors but discourage other-involved preventive behaviors. Regression analyses suggested that independent self-construal might not be associated with washing hands (*p*_*adj*_ = 0.30) or cleaning and disinfecting frequently touched surfaces (*p*_*adj*_ = 0.99), thus not supporting H3(a). In addition, contrary to H4(b), independent self-construal positively predicted three out of the five other-involved preventive behaviors, namely, keeping social distance with *b* = 0.07, *t*_(1720)_ = 3.52, and *p*_*adj*_ = 0.003; staying home with *b* = 0.09, *t*_(1720)_ = 4.33, and *p*_*adj*_ < 0.001; and avoiding public transportation with *b* = 0.10, *t*_(1720)_ = 4.14, and *p*_*adj*_ < 0.001. Independent self-construal was not associated with wearing face masks (*p*_*adj*_ = 0.99) or covering nose and mouth were coughing or sneezing (*p*_*adj*_ = 0.06). Therefore, H4 was not supported, and the reversed finding is discussed later in the article.

H5 was regarding the national differences in the health locus of control. The results showed that, compared to American participants, Chinese participants reported a lower level of health locus of control by internal factors, *b* = −1.51, *t*_(1724)_ = −22.25, and *p*_*adj*_ < 0.001; a higher level of health locus of control by powerful others, *b* = 0.68, *t*_(1724)_ = 10.09, and *p*_*adj*_ < 0.001; and a lower level of health locus of control by chance, *b* = −1.86, *t*_(1724)_ = −23.33, and *p*_*adj*_ < 0.001. Therefore, H5 was supported.

H6(a) and H6(b) predicted that the health locus of control by powerful others was positively associated with both self-focused and other-involved preventive behaviors. The results showed that the health locus of control by powerful others was positively related to cleaning and disinfecting frequently touched surfaces with *b* = 0.06, *t*_(1720)_ = 2.15, and *p*_*adj*_ = 0.03, but not necessarily washing hands (*p*_*adj*_ = 0.99), thus partially supporting H6(a). Participants' health locus of control by powerful others was positively associated with wearing face masks with *b* = 0.28, *t*_(1720)_ = 10.02, and *p*_*adj*_ < 0.001; keeping social distance with *b* = 0.06, *t*_(1720)_ = 3.15, and *p*_*adj*_ = 0.01; and staying home with *b* = 0.09, *t*_(1720)_ = 5.07, and *p*_*adj*_ < 0.001 but not necessarily covering nose and mouth when sneezing and coughing (*p*_*adj*_ = 0.33) or avoiding public transportation (*p*_*adj*_ = 0.99), partially supporting H6(b).

H7(a) and H7(b) proposed that the health locus of control by chance was negatively associated with both self-focused and other-involved preventive behaviors. Regression analyses revealed that health locus of control by chance was negatively related to engagement in washing hands with *b* = −0.10, *t*_(1720)_ = −6.43, and *p*_*adj*_ < 0.001 but not necessarily cleaning and disinfecting frequently touched surfaces (*p*_*adj*_ = 0.99), partially supporting H7(a). Similarly, the health locus of control by chance was negatively associated with wearing face masks with *b* = −0.06, *t*_(1720)_ = −2.75, and *p*_*adj*_ = 0.04; covering nose and mouth when sneezing and coughing with *b* = −0.10, *t*_(1720)_ = −6.49, and *p*_*adj*_ < 0.001; keeping social distance with *b* = −0.11, *t*_(1720)_ = −7.52, and *p*_*adj*_ < 0.001; staying home with *b* = −0.08, *t*_(1720)_ = −5.37, and *p*_*adj*_ < 0.001; and avoiding public transportation with *b* = −0.13, *t*_(1720)_ = −8.35, and *p*_*adj*_ < 0.001. Therefore, H7(b) was supported.

Finally, H8(a) and H8(b) predicted that health locus of control by internal factors would be positively associated with self-focused preventive behaviors but negatively associated with other-involved preventive behaviors. Supporting H8(a), regression analyses showed that health locus of control by internal factors was positively associated with washing hands with *b* = 0.12, *t*_(1720)_ = 6.50, and *p*_*adj*_ < 0.001 and cleaning and disinfecting frequently touched surfaces with *b* = 0.15, *t*_(1720)_ = 6.13, and *p*_*adj*_ < 0.001. In terms of other-involved preventive behaviors, health locus of control by internal factors was negatively related to wearing facemasks with *b* = −0.15, *t*_(1720)_ = −5.68, and *p*_*adj*_ < 0.001 but not necessarily keeping social distance (*p*_*adj*_ = 0.99), staying home (*p*_*adj*_ = 0.99), or avoiding public transportation (*p*_*adj*_ = 0.07). Contrary to the prediction, participants' health locus of control by internal factors was positively associated with engagement in covering nose and mouth when sneezing and coughing with *b* = 0.08, *t*_(1720)_ = 4.22, and *p*_*adj*_ < 0.001. Therefore, H8(b) was supported for only one out of the five other-involved preventive behaviors.

## 5 Discussion

This study identified significant national differences between Americans and Chinese people's engagement in seven preventive behaviors that are commonly recommended by public health authorities to battle a public health crisis. To theorize these national differences, we proposed two conceptual types of preventive behaviors, namely, self-focused and other-involved behaviors. The findings suggest that Americans engaged more in self-focused preventive behaviors (i.e., washing hands and disinfecting surfaces) than Chinese, whereas Chinese engaged more in the majority of the other-focused preventive behaviors (i.e., wearing face masks, keeping social distance, and staying home) than Americans. Additionally, Chinese participants reported greater engagement in other-involved preventive behaviors than self-focused behaviors, although we only observed non-significant differences between the two types of behaviors reported by American participants. Moreover, we found that these national differences can be explained by two psychological traits, namely, self-construal and health locus of control.

The distinction between self-focused and other-involved preventive behaviors, along with the identification of self-construal and the health locus of control as two individual-level mechanisms, contributed to the understanding of preventive behaviors for public health crises. The two-fold distinction of health preventive behaviors highlights the communal nature of many public health crises: while it is reasonable to emphasize the importance of self-focused preventive behaviors in managing many personal health issues, communal risks imposed by large-scale health crises require not only self-protection but also collective actions involving others' efforts (4). Previous research on health promotion has centered on self-focused behaviors such as smoking cessation, weight loss, and depression treatment [for a meta-analysis, see (33)]. Studies examining public health crises, such as Middle East respiratory syndrome or coronavirus, have primarily focused on how exposure and processing of health information contributed to risk perceptions and subsequent preventive behaviors [e.g., Choi et al. (34); Nazione et al. (35)]. However, these studies have often overlooked the conceptual or empirical differentiation of various preventive behaviors. Acknowledging the communal nature of some public health issues helps to connect the health communication research that focuses on individual behaviors with other social scientific research that highlights the interdependence among individuals, such as risk management/communication, group and intergroup processes, and social movements.

The two individual traits, namely, self-construal and health locus of control, help us further understand the diverse responses to public health crises. They serve as the individual-level mechanisms that partially explain the national differences in self-focused and other-involved preventive behaviors. While some established health prevention models (e.g., the Health Belief Model, (36); the Extended Parallel Process Model, (37) often do not consider culture-induced individual differences (38), our model and findings highlight the importance of such differences. The observed national differences between Americans and Chinese in the health locus of control not only exemplify the distinctive national cultures of the two societies but also mirror variances in their institutional healthcare frameworks (e.g., health institutions are predominantly state-owned in China, while privatized in the United States). These results highlight the importance of designing health campaigns and interventions tailored to the national culture as well as societal and institutional frameworks.

However, our data still left a few hypotheses unconfirmed and even reversed, demanding further explanations and investigations. First, it was surprising to find a positive association between independent self-construal and engagement in several other-involved preventive behaviors. One possible explanation is that, although other-involved preventive behaviors imply a greater awareness of interdependence compared to self-focused behaviors, these behaviors are not intrinsically altruistic and are still tied to self-interest. Therefore, it is possible that independent self-construal would also motivate other-involved preventive behaviors—especially if the individuals were aware of the self-interest in other-involved preventive behaviors. In other words, the awareness of self- vs. other-interest implied in preventive behaviors may be an important boundary condition of this part of our model and thus deserves further investigation.

Second, although the between-nation differences in the two types of preventive behaviors largely confirmed our hypotheses (H1a and b), the within-nation differences were less clear. While Chinese participants indeed reported greater engagement in other-involved behaviors compared to self-focused preventive behaviors (supporting H2b), the two types of preventive behaviors did not differ among American participants (rejecting H2a). One possible explanation could be that Americans may not differentiate between self-focused and other-involved preventive behaviors as much as their Chinese counterparts. Considering that both types of preventive behaviors were recommended and even reinforced by governments and health authorities, Americans might either accept these recommended preventive behaviors as a whole or completely reject them. By analyzing 12-week Twitter posts on mask-wearing, Rains et al. (33) found that public anger was not centered around the mask mandates as an obstacle to personal freedom; instead, mask-wearing was deemed as protection from harm. Moreover, the COVID-19 pandemic has been largely politicized and Americans have polarized opinions and attitudes regarding the perceived risk, trust in politicians to handle the pandemic, and trust in health authorities such as the World Health Organization (39) (40). Future studies should continue to examine the within-national variations in responses to the two types of preventive behaviors and how political ideology and partisanship account for these variations.

Finally, the proposed negative impact of health locus of control by internal factors on other-involved preventive behaviors was not observed. It is possible that, on the one hand, individuals with a higher locus of control by internal factors may perceive other-involved preventive behaviors to be less controllable by themselves and, thus, are discouraged from engaging in these behaviors. On the other hand, individuals may recognize the benefits of other-involved preventive behaviors even without others' cooperation and attempt to still engage. Future research should examine whether the two competing mechanisms could explain the absence of an association between the health locus of control by internal factors and engagement in other-involved preventive behaviors.

### 5.1 Practical implications

Our findings offer implications for designing communication interventions to maximize the impacts of health campaigns aimed at preventing a public health crisis. Instead of attempting to identify a one-size-fits-all solution, our study suggests that recovery from a public health crisis requires tailored interventions. Although national culture and its dimensions, such as individualism-collectivism, power distance, and indulgence, are presumably stable and universal within societies, the related individual traits are not. Traits including self-construal and health locus of control are situation-dependent and can be intervened through persuasion and other forms of communication. Policymakers and communication practitioners should take into account the underlying psychological mechanisms in designing intervention messages (41). For instance, highlighting interdependence in coping with communal risks in a public health crisis may evoke individuals' interdependent self-construal and motivate them to perform other-involved preventive behaviors. Similarly, messages that emphasize the roles of self and public health authorities in crisis containment may instill confidence in people to adopt both self-focused and other-involved preventive behaviors. Embedding such cultural beliefs in health campaigns and health message designs will help promote individuals' health perceptions and behaviors.

### 5.2 Limitations

This study has several limitations. First, although we have collected survey data from both the United States and China, the data were cross-sectional, limiting our ability to test for causal relationships. Second, this study used online survey tools to recruit participants. Considering the digital divide and possible self-selection bias, the participants recruited from the online survey panel may not represent the entire population of each country. Third, while the study emphasized individual-level psychological mechanisms for preventive behaviors between the United States and China, cultural dimensions were incorporated as umbrella concepts without an empirical assessment of their relationships with individual behaviors. The dynamic and complex nature of culture may not be fully captured by the variables being examined in the current study. Fourth, the scale to measure the health locus of control exhibited relatively lower reliability within the Chinese sample. Since the health locus of control has rarely been measured with Chinese participants and no valid Chinese version of the measure has been developed, the scale utilized in this study was an appropriate choice. Finally, the dichotomization of self-focused and other-involved behaviors may simplify the complex interplay between individual and communal health practices, as certain preventive behaviors may be related to both types.

### 5.3 Future directions

We propose several directions to continue this line of research. First, future studies should adopt longitudinal designs to establish causal relationships and monitor changes in individuals' perceptions, social norms, and behaviors in response to public health crises. In relation to the design, future studies should use more comprehensive and sophisticated sampling techniques to acquire national representative samples to compare the national differences in response to public health crises.

Future studies should further explore additional cultural factors manifested at various levels, including individual, community, organizational, and societal levels. Factors such as risk perceptions, social integration, political views, and governmental reinforcements of preventive behaviors potentially contribute to cultural differences in responses to a public health crisis. For instance, while lockdowns were implemented in both China and the United States during the data collection period, the level of reinforcement strictness varied between the two countries, resulting in potential differences in the adoption of preventive behaviors.

Finally, future studies should continue to extend and apply the self-focused vs. other-involved dichotomy of preventive behaviors. Although the current study only provides preliminary findings regarding this dichotomized construct, future studies could explore its applicability to other types of preventive behaviors aimed at minimizing individual and communal risks and assess the boundary of such categorizations.

## 6 Conclusion

By using two large-scale cross-sectional surveys conducted in the United States and China, this study found that, when facing public health crises, Americans engaged more in self-focused preventive behaviors, while Chinese engaged more in other-involved preventive behaviors. Within each country, Americans engaged more in self-focused than other-involved preventive behaviors, while Chinese engaged more in other-involved than self-focused preventive behaviors. The national differences in people's engagement in preventive behaviors between the United States and China can be partially explained by individuals' independent and interdependent self-construals, as well as their health locus of control. The culture-sensitive approach exemplified a meaningful and fruitful way to advance our understanding of public health preventive behaviors in the increasingly globalized world.

## Data availability statement

The original contributions presented in the study are included in the article/Supplementary material, further inquiries can be directed to the corresponding author.

## Ethics statement

The studies involving humans were approved by the College of Journalism and Communication, Renmin University of China. The studies were conducted in accordance with the local legislation and institutional requirements. The participants provided their written informed consent to participate in this study.

## Author contributions

WP: Writing – original draft, Writing – review & editing. WL: Writing – original draft, Writing – review & editing. BF: Writing – original draft, Writing – review & editing. SL: Writing – original draft, Writing – review & editing.
