# Supplementary material for: Explaining differences in self-focused and other-involved public health preventive behaviors between the US and China: the role of self- construal and health locus of control
Source: Front Public Health. 2024 Feb 22;12:1321506. doi: 10.3389/fpubh.2024.1321506 (PMC10917930; doi:10.3389/fpubh.2024.1321506)
Supplement: Supplementary file 1 [file Table_1.docx]

**Supplementary Materials**

**Table S1. Demographics of the American Sample (N = 888)**

| Demographics | | | American Participants |
| --- | --- | --- | --- |
| Female, n (%) | | | 422 (47.5) |
| Age (years), mean (SD) | | | 37 (11.94) |
| Marital status, n (%) | | |  |
|  | Single | | 396 (44.6) |
|  |  | Single | 334 (37.6) |
|  |  | Widowed | 7 (0.8) |
|  |  | Divorced | 45 (5.1) |
|  |  | Separated | 10 (1.1) |
|  | Married/domestic partnership | | 492 (55.4) |
| Employment status, n (%) | | |  |
|  | Working | | 726 (81.7) |
|  |  | Self-employed | 121 (13.6) |
|  |  | Working full time for wages | 523 (58.9) |
|  |  | Working part time for wages | 82 (9.2) |
|  | Not working | | 162 (18.3) |
|  |  | Out of work | 84 (9.5) |
|  |  | Not able to work or disabled | 12 (1.4) |
|  |  | Retired | 25 (2.8) |
|  |  | Other | 41 (4.6) |
| Education level, n (%) | | |  |
|  | Less than high school degree | | 4 (0.5) |
|  | High school graduate | | 54 (6.1) |
|  | Some college but no degree | | 135 (15.2) |
|  | Associate degree in college | | 92 (10.4) |
|  | Bachelor’s degree in college | | 404 (45.5) |
|  | Master’s degree | | 167 (18.8) |
|  | Doctoral degree | | 17 (1.9) |
|  | Professional degree (JD, MD) | | 15 (1.7) |
| Household income (US $), n (%) | | |  |
|  | <10,000 | | 35 (6.3) |
|  | 10,001-20,000 | | 50 (9.0) |
|  | 20,001-40,000 | | 105 (18.9) |
|  | 40,001-60,000 | | 109 (19.6) |
|  | 60,001-80,000 | | 106 (19.1) |
|  | 80,001-100,000 | | 61 (11.0) |
|  | 100,001-120,000 | | 36 (6.5) |
|  | >120,000 | | 53 (9.5) |
| Race, n (%) | | |  |
|  | White | | 607 (68.4) |
|  | Black or African American | | 106 (11.9) |
|  | Hispanic or Latino American | | 45 (5.1) |
|  | Asian or Asian American | | 89 (10.0) |
|  | Other | | 41 (4.6) |

**Table S2. Demographics of the Chinese Sample (N = 844)**

| Demographics | | | Chinese Participants |
| --- | --- | --- | --- |
| Female, n (%) | | | 469 (55.6) |
| Age (years), mean (SD) | | | 31.71 (8.4) |
| Marital status, n (%) | | |  |
|  | Single | | 274 (32.4) |
|  |  | Single | 262 (31) |
|  |  | Widowed | 2 (0.2) |
|  |  | Divorced | 9 (1.1) |
|  |  | Separated | 1 (0.1) |
|  | Married/domestic partnership | | 570 (67.5) |
| Employment status, n (%) | | |  |
|  | Working | | 766 (90.8) |
|  |  | Self-employed | 62 (7.3) |
|  |  | Working full time for wages | 679 (80.5) |
|  |  | Working part time for wages | 25 (3.0) |
|  | Not working | | 78 (9.1) |
|  |  | Out of work | 8 (0.9) |
|  |  | Not able to work or disabled | 1 (0.1) |
|  |  | Retired | 7 (0.8) |
|  |  | Other | 62 (7.3) |
| Education level, n (%) | | |  |
|  | No formal education or some elementary school | | 2 (0.2) |
|  | Junior high school graduate | | 12 (1.4) |
|  | Senior high school graduate | | 31 (3.7) |
|  | Secondary or vocational high school | | 17 (2.0) |
|  | Associate degree | | 122 (14.5) |
|  | Bachelor’s degree | | 593 (70.3) |
|  | Master’s degree | | 63 (7.5) |
|  | Doctoral degree | | 4 (0.5) |
| Monthly income (Chinese yuan), n (%) | | |  |
|  | No income | | 56 (6.6) |
|  | Less than 1000 | | 18 (2.1) |
|  | 1001-3000 | | 82 (9.7) |
|  | 3001-5000 | | 175 (20.7) |
|  | 5001-8000 | | 250 (29.6) |
|  | 8000-10,000 | | 120 (14.2) |
|  | 10,001-15,000 | | 89 (10.5) |
|  | 15,001-20,000 | | 32 (3.8) |
|  | 20,001-50,000 | | 21 (2.5) |
|  | More than 50,000 | | 1 (0.1) |

**Table S3. Survey Items**

|  | English Items | Chinese Items （中文） |
| --- | --- | --- |
| Self-focused preventive behaviors | 1. Wash hands regularly for 20 seconds, with soap and water or alcohol-based hand rub 2. Clean and disinfect frequently touched surfaces such as doorknobs, phones, and keyboards | 1. 用肥皂或洗手液洗手不少于20秒 2. 每天对经常接触的物品表面进行消毒（比如门把手、手机、电脑键盘等） |
| Other-involved preventive behaviors | 1. Wear a facemask in public even if I am not sick 2. Cover nose and mouth with a disposable tissue or flexed elbow when cough or sneeze 3. Keep safe social distance with others 4. Stay home 5. Avoid using public transportation | 1. 在公众场合戴口罩 2. 打喷嚏时用纸巾或手肘遮住口鼻 3. 与他人保持一定的距离 4. 呆在家里 5. 不搭乘公共交通工具 |
| Independent self-construal | 1. My personal identity, independent from others, is important to me 2. I prefer to be self-reliant rather than dependent on others 3. I act as a unique person, separate from others 4. It is important for me to act as an independent person 5. I enjoy being unique and different from others | 1. 对我来说,拥有自己的个性和独立非常重要 2. 我更倾向于自力更生，而不是依靠他人 3. 我是与别人不同的独立个体 4. 拥有独立的人格对我来说很重要 5. 我很享受与众不同的感觉 |
| Interdependent self-construal | 1. My relationships with my friends and family are more important than my personal accomplishments 2. I am careful to maintain harmony among my friends and family 3. I would sacrifice my self-interest for the benefit of my family and friends 4. I try to meet the demands of my group, even if means controlling my own desires 5. It is important to consult close friends and get their ideas before making decisions | 1. 我与家人朋友的关系比我的个人成就更重要 2. 我很注重与家人朋友和谐共处 3. 我会为了家人朋友牺牲自己的利益 4. 我会为了满足我所在群体的利益而控制自己的欲望 5. 在做决定前征求好友的意见对我来说非常重要 |
| Internal-health locus of control | 1. If I fall ill with the virus, I believe I can recover on my own without going to the hospital 2. If I contract the virus, my health will depend on how well I look after myself 3. I can pretty much stay healthy by taking good care of myself | 1. 如果我感染了新冠病毒，我相信我不用去医院就可以自愈 2. 如果我感染了新冠病毒，我能否康复主要取决于我能否很好地照顾自己 3. 如果我好好照顾自己，我就能保持健康的状态 |
| Powerful others-health locus of control | 1. If I fall ill with the virus, I believe the health care system will help me recover 2. If I contract the virus, its impact on my health will depend on the quality of treatment I receive from physicians 3. If I fall ill with the virus, health professionals’ treatment and care will decide whether I recover from the illness | 1. 如果我感染了新冠病毒，医疗机构会帮助我康复 2. 如果我感染了新冠病毒，我能否痊愈取决于现有的医疗水平 3. 如果我感染了新冠病毒，医护人员的治疗和照顾会决定我能否痊愈 |
| Chance-health locus of control | 1. I often feel that no matter what I do, if I am going to fall ill with the virus, it is meant to be like that 2. It seems as if my health mostly depends on sheer coincidence 3. If I am NOT infected with the virus, it is because I am lucky 4. If I am ill, I must let nature take its cause | 1. 我觉得无论我做什么，是否会感染新冠病毒是命中注定的 2. 我的健康状况主要由偶然因素决定 3. 如果我没有感染新冠病毒，是因为我运气好 4. 如果我感染了新冠病毒，我会顺其自然 |
